# Systematic Intravenous Administration of Autologous Mesenchymal Stem Cells Is Safe

**DOI:** 10.3390/jcm13237460

**Published:** 2024-12-07

**Authors:** Takaaki Matsuoka, Takaaki Itohara, Yurie Hara, Nana Kobayashi

**Affiliations:** Omotesando HELENE Clinic, 5-9-15-3F, Minami Aoyama, Minato City, Tokyo 1070062, Japan

**Keywords:** mesenchymal stem cells (MSC), adipose-derived stem cells, cardiovascular diseases, cerebrovascular diseases, major adverse cardiac and cerebrovascular events (MACCE), dose variability, survival analysis, Cox proportional hazards model, Kaplan-Meier analysis, arteriosclerosis

## Abstract

**Background**: Mesenchymal stem cells (MSCs) have drawn significant attention for their regenerative potential and therapeutic applicability across a range of conditions, including cardiovascular diseases and age-related frailty. Despite extensive preclinical studies, there remain gaps in understanding the long-term safety and efficacy of MSC therapy in humans. This study aimed to assess the safety of intravenous MSC administration, evaluate the mean major adverse cardiac and cerebrovascular event (MACCE)-free period, and identify potential risk factors for MACCE development in patients receiving MSC therapy for various indications. **Methods**: A retrospective observational study was conducted on 2504 patients (mean age: 54.09 ± 11.65 years) who received intravenous adipose-derived MSC (AD-MSC) therapy between October 2014 and December 2023 at the Omotesando Helene Clinic, Tokyo, Japan. Patients received MSC doses ranging from 100 million to 2 billion cells, with the majority receiving 1–2 billion cells per treatment. Statistical analyses included multivariate Cox proportional hazards regression and Kaplan–Meier survival analysis to evaluate MACCE risk factors and event-free duration. **Results:** Over the follow-up period, the MACCE rate was exceptionally low at 0.2%. Multivariate analysis identified age as a significant risk factor for MACCE (hazard ratio: 1.127; 95% CI: 1.0418–1.219; *p* = 0.0029), while sex and MSC dose showed no significant association. Minor adverse events occurred in 0.8% of patients, with no severe adverse events reported. The study found MSC therapy to be safe, with a low adverse event rate and minimal risk of MACCE. **Conclusions**: This study demonstrates the safety of intravenous MSC therapy in a large cohort of patients, with a low incidence of MACCE and minimal adverse effects. Age was the only significant predictor of MACCE risk. Further prospective randomized studies are needed to validate these findings and explore the potential of MSC therapy in reducing MACCE risk and improving clinical outcomes across diverse indications.

## 1. Introduction

Mesenchymal stem cells (MSCs) are somatic stem cells with potential for self-renewal, immunomodulation, and multilineage differentiation [1]. The potential therapeutic applicability of stem cells has been empirically investigated for a range of conditions, such as acute myocardial infarction [2], inflammatory bowel disease [3], traumatic spinal cord injury [4], diabetes melluitus [5], and ageing frailty [6,7]. The beneficial effects of MSCs are thought to be caused not only by engraftment and differentiation, but also by the secretion of biologically active cells that exert beneficial effects on other cells [1]. The trophic activities of MSCs include anti-fibrotic, immunomodulatory, anti-apoptotic, chemoattractive, neurogenic, and angiogenic properties [1].

A recent article noted that, as of April 2023, despite 1120 registered clinical trials for MSC therapy worldwide, only 12 MSC therapies have been approved by regulatory agencies for commercial application [8]. There are recognised challenges in translating promising preclinical findings of MSC therapies into clinical trial results and then to the clinic [8], but the immense potential for MSC therapies to transform the management of a wide range of conditions offers great promise to the medical and scientific communities worldwide.

Lifestyle-related diseases, such as diabetes, hypertension, and hyperlipidaemia, can damage the endothelium of major blood vessels, leading to the development of major adverse cardiac and cerebrovascular events (MACCE), ultimately leading to death [9,10]. Although animal studies have already shown that the intravenous administration of stem cells promotes vascular endothelial regeneration [11], clinical research has confirmed that intravenous administration in humans can regenerate major blood vessels to a clinically meaningful extent, leading to reduced long-term complications and mortality rate. To our knowledge, no large clinical study has evaluated the risk factors for MACCEs in patients receiving MSC therapy for various indications. This study aimed to evaluate the mean MACCE-free period and potential risk factors for MACCE development in a population of patients receiving MSC therapy for various indications.

### Research Background

Mesenchymal stem cells (MSCs) have garnered significant attention in regenerative medicine due to their unique capabilities. These somatic stem cells, derived from mesenchymal tissues such as bone marrow, adipose tissue, and umbilical cords, possess self-renewal abilities, immunomodulatory properties, and the potential to differentiate into various cell lineages. Adipose-derived MSCs (AD-MSCs), in particular, have become a preferred source in clinical applications due to their ease of harvest, high yield, and robust proliferation capabilities in vitro. Their regenerative potential has been extensively investigated in treating a variety of conditions, including cardiovascular diseases, inflammatory bowel diseases, neurological disorders, diabetes mellitus, and age-related frailty.

Despite promising preclinical data and a growing body of clinical studies, the pathway from bench to bedside remains fraught with challenges. Translational barriers include variability in MSC preparation methods, lack of standardized dosing protocols, and uncertainties regarding their long-term safety and efficacy. Furthermore, regulatory agencies worldwide have approved only a limited number of MSC therapies, emphasizing the need for robust data to support widespread clinical adoption. Addressing these gaps is essential to unlock the full potential of MSC-based therapies.

Lifestyle-related diseases, such as diabetes, hypertension, and hyperlipidemia, contribute significantly to vascular endothelial damage, which is a primary driver of major adverse cardiac and cerebrovascular events (MACCE). While animal studies have demonstrated that intravenous MSC administration promotes vascular repair and endothelial regeneration, large-scale clinical studies evaluating the safety and efficacy of MSC therapy in humans are sparse. This study aims to fill this gap by investigating MACCE-free periods and identifying potential risk factors for MACCE in a diverse cohort of patients receiving MSC therapy.

## 2. Materials and Methods

### 2.1. Patient Selection and Ethics

This retrospective observational cohort study was conducted at the Omotesando Helene Clinic in Tokyo, Japan, from October 2014 to December 2023. Patients eligible for inclusion were adults (≥20 years old) who had received intravenous AD-MSC therapy and provided written informed consent. The study protocol adhered to the ethical principles outlined in the Declaration of Helsinki and received approval from the institutional review board (Helene Ethical Committee; approval number: HCS-20140601).

To ensure the appropriateness of MSC administration, each patient underwent a thorough medical evaluation by a licensed physician. The exclusion criteria included individuals deemed unsuitable for MSC therapy due to medical or logistical reasons, as determined by the principal investigator.

### 2.2. MSC Culturing and Preparation

AD-MSCs were isolated from adipose tissue using established protocols compliant with Good Manufacturing Practice (GMP) guidelines. The adipose tissue was processed in a sterile environment to extract stromal vascular fractions, which were then expanded in culture to obtain sufficient cell numbers. Quality control measures included viability assays, sterility tests, and flow cytometric analysis to confirm the expression of key MSC markers (e.g., CD73, CD90, CD105) and the absence of hematopoietic markers (e.g., CD34, CD45).

Each MSC dose was tailored to the patient’s clinical condition, with doses ranging from 100 million to 2 billion cells dissolved in 200 mL of 0.9% saline. The preparation process ensured that the cells maintained their viability and potency without inducing unwanted differentiation or genetic modifications. Patients received intravenous infusions in a dedicated outpatient treatment room under close medical supervision.

### 2.3. Study Design and Ethical Approval

In this single-centre, observational, retrospective cohort study, written informed consent was obtained from all patients prior to stem cell administration. The study was conducted in accordance with the Declaration of Helsinki and approved by the Institutional Review Board of our institution (Helene Ethical Committee; approval number: HCS-20140601).

### 2.4. Treatment

In patients who received multiple doses, the date of administration was calculated from the date of the first dose. The dose of mesenchymal stem cells administered to each patient was not fixed and ranged from 100 million to 2 billion cells, with most patients receiving 1 or 2 billion cells. Dosing decisions were based on the physician’s diagnosis.

Each dose of mesenchymal stem cells was administered as a single intravenous dose and was not split. Culture-grown MSCs were dissolved in 200 mL of 0.9% saline and administered intravenously in an outpatient treatment room. The total time required for the administration of MSCs was approximately 1 h. Treatment was completed after 30 min, after which the medical staff monitored the patients for any abnormalities in their physical condition and confirmed that there were no abnormal vital signs or other irregularities. Post-administration monitoring was conducted by telephone or other means on the following day to check for complications during the acute phase.

### 2.5. Follow-Up

Clinical follow-up included medical record reviews, telephone, email conversations, and administration of questionnaires to patients, their families, and caregivers. Follow-Up Protocol is as follows: Following MSC administration, patients were monitored for immediate adverse events for a minimum of 30 min post-infusion. Additional follow-up included regular medical record reviews, telephone consultations, and questionnaires to assess long-term outcomes. Data on side effects, MACCE incidence, and general health status were systematically collected.

### 2.6. Endpoints

The primary endpoint of the study was the MACCE rate during the follow-up period, defined as a composite of all-cause mortality and non-fatal myocardial infarction (MI)/non-fatal stroke. The secondary endpoints included the occurrence of minor side effects such as fever, dizziness, insomnia, pruritus, and nausea.

### 2.7. Statistical Analyses

Categorical variables were represented as frequencies and percentages. Univariate Cox regression analyses were performed for each predictor to identify individual associations with MACCE risk. Multivariate Cox proportional hazard regression models were used to assess the associations between various factors and MACCE. Kaplan–Meier survival analysis was performed to evaluate the probability of MACCE-free status during the follow-up period. Patients who were alive but could not be followed up were censored as dropouts. ROC analysis was performed to test the classification ability to discriminate MACCE status among predictors. The classification ability was tested using AUC analysis. The classification ability of the model was evaluated using Gini’s Index and Maximum K-S value, and the Kolmogorov–Smirnov test was performed to determine the optimal threshold value for predictors that could provide the best classification for MACCE status.

However, power calculations were not performed. The *p*-value threshold for statistical significance was set at *p* < 0.05. Statistical analyses were performed using R version 4.3.2 (R Foundation for Statistical Computing, Vienna, Austria) and Microsoft Excel (Microsoft Corp., Redmond, WA, USA), except for the ROC analysis, which was performed using SPSS (version 27.0 (IBM Corp., Armonk, NY, USA)).

## 3. Results

### 3.1. Study Population and Setting

Study population characteristics are summarised in Table 1. This study included 2504 patients who received intravenous stem cells at the Omotesando Helene Clinic between October 2014 and December 2023. The average age of patients was 54.09 ± 11.65 years. Among the 2504 patients, 1276 (50.96%) were female and 1225 (48.92%) were male; the sex details of three (0.12%) patients were missing. The total number of intravenous doses of mesenchymal stem cells administered in this group was 4431, and the mean dose of stem cells received per participant was 1.49 ± 0.75 billion cells. The average length of follow-up was 15.88 ± 26.85 months. The nationality distribution of patients was as follows: 72% (*n* = 1802) were Chinese, 16% (*n* = 402) Vietnamese, 6% (*n* = 152) Japanese, and 6% (*n* = 148) others, almost all of whom were from East or Southeast Asia.

### 3.2. Statistical Analysis

**Figure 1:**
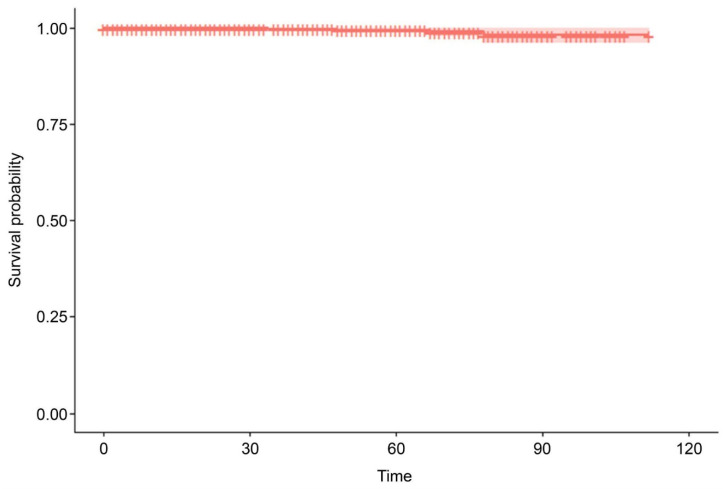
Kaplan–Meier survival curve showing the major adverse cardiac and cerebrovascular events (MACCE)-free status over time.

**Figure 2:**
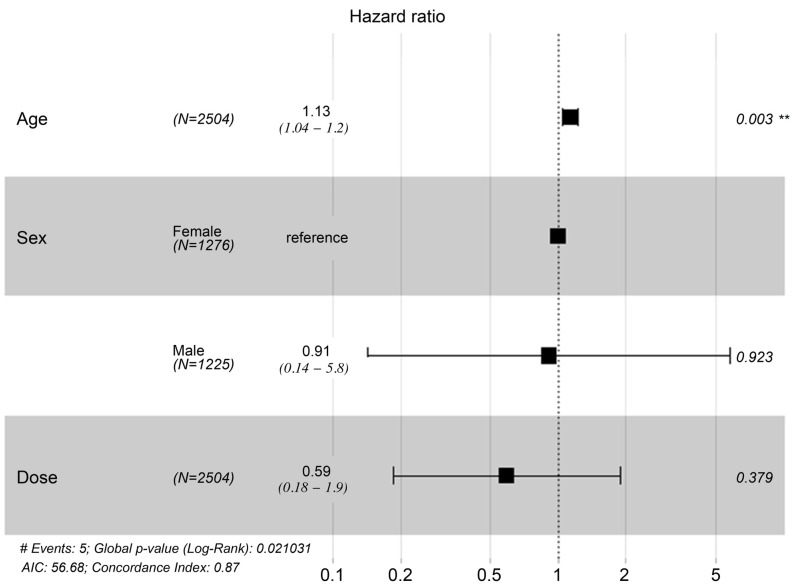
Hazard ratio and 95% confidence interval (CI). Forest plot evaluation of variables predicting MACCE-free status in the study population. Error bars represent the 95% confidence intervals. **: *p*-value less than 0.05 was considered statistically significant.

The indications for MSC administration varied (Table 1), but over two-thirds (*n* = 1700, 67.89%) received MSC therapy as an anti-aging therapy, of whom 712 were followed up. There were five MACCE and deaths during the mean observation period of 112 months (standard error = 0.435; Table 2 and Figure 1), including four deaths (one due to coronavirus disease 2019-related pneumonia, one due to an unrelated incident, one due to natural causes [old age], and one due to stroke) and an additional case of non-fatal stroke. This represents an MACCE rate of 0.20% during the follow-up period. In univariate Cox proportional hazards regression analysis, an analysis of age, gender, and MSC dose revealed that only age was significantly associated with a risk of MACCE (hazard ratio = 1.124; 95% confidence interval = 1.04, 1.214; *p* = 0.0031; Table 3). Specifically, the risk of MACCE increases by 1.124 per unit increase in age. In a multivariate Cox proportional hazards regression analysis including the same three variables, age remained significantly associated with MACCE risk (hazard ratio = 1.127; 95% confidence interval = 1.0418–1.219; *p* = 0.0029; Table 4 and Figure 2), such that the risk of MACCE increased by 1.127 with each unit increase in age. Sex and MSC dose remained non-significant in multivariate analysis.

**Figure 3:**
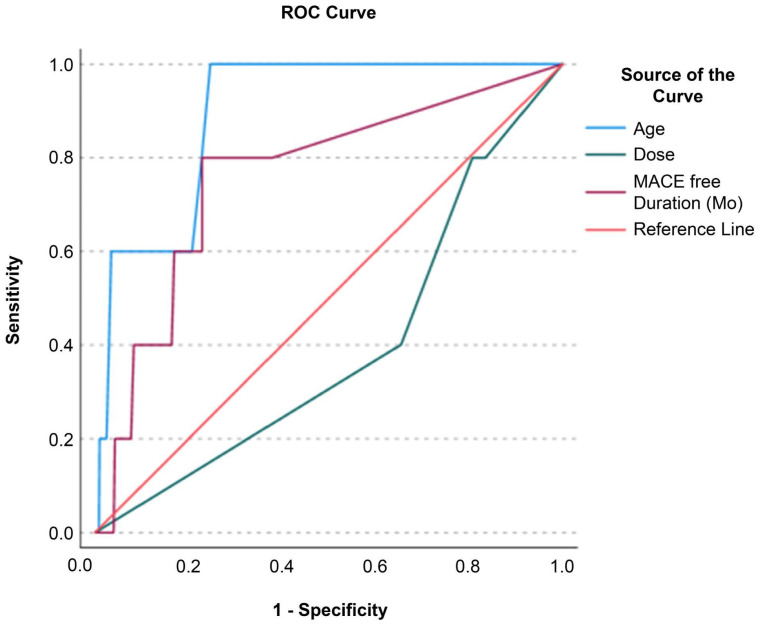
Receiver operating characteristic curve. The diagonal red line represents the reference line.

Receiver operating characteristic (ROC) analysis was performed to evaluate which of the variables age, MSC dose, and MACCE-free duration facilitated the classification of MACCE status (event vs. no event; Figure 3). Age and MACCE-free duration were excellent for classifying MACCE status, with area under the curve (AUC) values of 0.895 (*p* < 0.001, 95% confidence interval = 0.807–0.984) and 0.759 (*p* = 0.019, 95% confidence interval = 0.542–0.976), respectively. MSC dose, with an AUC of 0.394 (*p* = 0.377; 95% confidence interval = 0.158, 0.630), was not a significant predictor. Classifier evaluation metrics confirmed the superiority of age (Gini Index = 0.791; maximum Kolmogorov–Smirnov metric = 0.754; Kolmogorov–Smirnov metric cut-off = 61.50) and MACCE-free duration (Gini Index = 0.518; maximum Kolmogorov–Smirnov metric = 0.571; Kolmogorov–Smirnov metric cut-off = 33.50) over the MSC dose (Gini Index = −0.213; maximum Kolmogorov–Smirnov metric = 0.000; Kolmogorov–Smirnov metric cut-off = 3.00).

### 3.3. Adverse Effects

Most MSC therapy recipients (*n* = 2484, 99.2%) experienced no severe adverse effects (Table 1). The most common adverse event was insomnia (*n* = 10, 0.4%), followed by disrupted menstrual cycles (*n* = 4, 0.16%) and dizziness (*n* = 2, 0.08%). Importantly, none of the adverse events were severe.

## 4. Discussion

This large, retrospective study of 2504 individuals who received MSC therapy for various indications showed that stem cell therapy is safe and associated with a very low risk of major adverse cardiac and cerebrovascular events over an average follow-up period of over 9 years. Multivariate analysis revealed that age was significantly associated with MACCE risk, and an AUC analysis of an ROC curve identified age and MACCE-free duration as significant predictors of MACCE status. Neither sex nor MSC dose was associated with MACCE risk. The adverse event rate was low (*n* = 20/2504, 0.8%), and none of the reported adverse events were severe.

There has been great interest in the use of MSC therapies to treat a wide range of medical conditions [2,3,4,5], as well as the effects of aging frailty [6,7]. Inflammation plays a significant role in the pathogenesis of aging-related frailty and is referred to in this context as “inflammaging” [12]. Inflammation is known to accelerate diseases associated with aging, including cardiovascular and cerebrovascular diseases that constitute MACCE [12]. Evidence from a phase II trial of patients diagnosed with frailty administered intravenous human allogeneic MSCs found the treatment to be safe and to improve physical performance, as well as inflammatory biomarkers, including serum tumour necrosis factor alpha levels [13]. In the current study, over two-thirds of the participants received MSC therapy as an anti-aging therapy, which holds promise as an application of MSC therapy to improve global health outcomes.

The current study had some limitations. First, its retrospective nature increases the risk of bias in the data collected; further prospective studies to corroborate our findings are warranted. Second, data on a matched control group are not available to demonstrate the potential MACCE-reducing benefits of MSC therapy. Future prospective studies should incorporate such a control group matched at baseline for risk factors associated with the development of MACCE, including cardiovascular risk factors [9].

In conclusion, our study showed that stem cell therapy is safe in a large cohort of individuals and is associated with a low rate of major cardiac and cerebrovascular events. Further prospective randomized studies are warranted to confirm that MSC therapy reduces MACCE events relative to placebo in age- and cardiovascular risk-matched controls.

## 5. Future Research Directions

### 5.1. Comparative Studies with Control Groups

One limitation of this study is the absence of a control group, which restricts the ability to directly attribute observed benefits to MSC therapy. Future studies should incorporate randomized controlled trials (RCTs) with matched control groups to provide robust evidence of the therapeutic effects of MSCs. These groups should be matched for baseline cardiovascular risk factors, age, and comorbidities to eliminate confounding variables.

### 5.2. Investigating Cellular Mechanisms and Biomarkers

While the therapeutic effects of MSCs are well documented, the precise mechanisms remain incompletely understood. MSCs are known to exert their effects primarily through paracrine signaling, releasing bioactive molecules such as cytokines, growth factors, and extracellular vesicles. Future research should focus on characterizing these secreted factors and their role in modulating inflammation, angiogenesis, and fibrosis. Biomarker studies could also identify patient populations most likely to benefit from MSC therapy, paving the way for more targeted and personalized approaches.

### 5.3. Long-Term Safety and Efficacy Studies

Although this study demonstrated a low adverse event rate, the long-term safety profile of MSC therapy requires further investigation. Multi-center longitudinal studies with extended follow-up periods are needed to assess the durability of therapeutic benefits and the potential risks of repeated MSC infusions.

## 6. Possible Applications (Comprehensive Expansion)

### 6.1. Anti-Aging Therapies

Age-related frailty and chronic inflammation, collectively referred to as “inflammaging”, are significant contributors to morbidity and mortality worldwide. MSCs have shown promise in mitigating these effects by modulating systemic inflammation and enhancing regenerative processes. In this study, over two-thirds of patients received MSC therapy for anti-aging purposes, highlighting its potential as a preventive strategy for age-associated diseases. Future research should explore the integration of MSC therapy into broader anti-aging regimens, including lifestyle modifications and pharmacological interventions.

### 6.2. Cardiovascular and Cerebrovascular Applications

Given the role of endothelial dysfunction in the pathogenesis of MACCE, MSC therapy offers a novel approach to vascular repair. By promoting endothelial regeneration and reducing inflammation, MSCs could serve as an adjunctive therapy for patients at high risk of cardiovascular events. Personalized treatment protocols, incorporating patient-specific risk profiles and biomarkers, could further enhance outcomes in this domain.

### 6.3. Advancing Personalized Medicine

The heterogeneity of patient responses to MSC therapy underscores the need for a personalized approach. Advances in omics technologies (e.g., genomics, proteomics, metabolomics) could enable the identification of predictive biomarkers for therapeutic success. This would allow clinicians to tailor MSC doses and treatment regimens to individual patient needs, maximizing efficacy while minimizing risks.

### 6.4. Expanding Therapeutic Indications

Beyond cardiovascular and anti-aging applications, MSCs hold promise for a wide range of conditions, including autoimmune diseases, neurodegenerative disorders, and metabolic syndromes. Ongoing research into the immunomodulatory and trophic effects of MSCs may uncover new indications, broadening their clinical utility and impact on global health outcomes.

## 7. Conclusions

This study provides robust evidence supporting the safety of intravenous administration of adipose-derived mesenchymal stem cells (AD-MSCs) in a large cohort of 2504 patients over a mean follow-up period of approximately nine years. The incidence of major adverse cardiac and cerebrovascular events (MACCE) was exceptionally low (0.2%), with age being the only significant predictor of MACCE risk. Neither sex nor MSC dose was associated with MACCE risk, highlighting the reliability of this therapeutic approach across diverse patient demographics.

Furthermore, the study observed a low overall adverse event rate (0.8%), with no severe adverse effects reported, reinforcing the clinical safety profile of MSC therapy. The findings also underline the therapeutic potential of MSCs in anti-aging applications, which constituted the majority of indications in this cohort.

Despite these promising results, the retrospective nature of the study and the absence of a matched control group limit the ability to establish causality. Future prospective, randomized controlled trials are essential to confirm these findings and explore the efficacy of MSC therapy in reducing MACCE and other long-term complications.

In conclusion, this study supports the potential role of MSC therapy as a safe and innovative approach for managing age-related frailty and reducing the burden of cardiovascular and cerebrovascular events. Further research is warranted to optimize treatment protocols and expand therapeutic applications, ensuring the effective integration of MSC-based therapies into clinical practice.

## Figures and Tables

**Table 1:** Patient characteristics.

Variables	Subcategory	Number of Patients (%)
Sex	Female	1276 (50.96%)
	Male	1225 (48.92%)
	Unknown	3 (0.12%)
Age (years)	Mean ± SD	54.09 ± 11.65
	Median (Min, Max)	54 (21, 104)
MSC IV Dose (in billions)	Mean ± SD	1.49 ± 0.75
	Median (Min, Max)	2 (0.1, 2)
Indications for MSC therapy	Allergy	23 (0.92%)
	ALS	4 (0.16%)
	AMI	60 (2.40%)
	AMI/Brain stroke	1 (0.04%)
	AMI/DM	7 (0.28%)
	AMI/DM/HT/HL	1 (0.04%)
	Anti-aging	1700 (67.89%)
	Arrythmia	8 (0.32%)
	Arteriosclerosis	1 (0.04%)
	Asthma	16 (0.64%)
	Atherosclerosis	51 (2.04%)
	Brain stroke	35 (1.40%)
	Brain stroke/renal failure	1 (0.04%)
	Brain stroke/AMI	1 (0.04%)
	Brain stroke/DM	1 (0.04%)
	Cancer	10 (0.40%)
	Cancer/DM	1 (0.04%)
	Cardiomyopathy	2 (0.08%)
	CNS disorder	1 (0.04%)
	Chronic obstructive pulmonary disease	2 (0.08%)
	Chronic obstructive pulmonary disease/AMI	1 (0.04%)
	Depression	5 (0.20%)
	DM	168 (6.71%)
	DM/arrythmia/hepatitis	1 (0.04%)
	DM/HT	1 (0.04%)
	DM/Renal failure	1 (0.04%)
	Emphysema	1 (0.04%)
	Heart valve disorder	3 (0.12%)
	Hepatitis	93 (3.71%)
	HL	116 (4.63%)
	HT	43 (1.72%)
	HT/DM	1 (0.04%)
	HT/Hepatitis	1 (0.04%)
	HT/HL	2 (0.08%)
	Insomnia	51 (2.04%)
	Infertility	2 (0.08%)
	Leukaemia	1 (0.04%)
	Long COVID	1 (0.04%)
	Mental disorder	2 (0.08%)
	Pancreatitis	1 (0.04%)
	Parkinson’s disease	6 (0.24%)
	Post cancer	17 (0.68%)
	Post-cancer surgery	1 (0.04%)
	Post pancreatitis	1 (0.04%)
	Renal failure	4 (0.16%)
	System lupus erythematosus	4 (0.16%)
	Type-1 diabetes	1 (0.04%)
	Thyroid disease	45 (1.80%)
	Tuberculosis	4 (0.16%)
Side effects	Disrupted menstrual cycles	4 (0.16%)
	Dizziness	2 (0.08%)
	Fatigue	1 (0.04%)
	Hives	1 (0.04%)
	Insomnia	10 (0.4%)
	Itch for 1 week	1 (0.04%)
	Psoriasis flare-up	1 (0.04%)
	None	2484 (99.2%)
MACCE-free duration (months)	Mean ± SD	15.88 ± 26.85
	Median (Min, Max)	0 (0, 112)

Abbreviations: ALS, amyotrophic lateral sclerosis; AMI, acute myocardial infarction; DM, diabetes mellitus; HT, hypertension; HL, hyperlipidaemia; MACCE, major adverse cardiac and cerebrovascular events; MSC, mesenchymal stem cells; SD, standard deviation.

**Table 2:** Mean MACCE-free time.

n	Events	Mean	SE (Mean)
2504	5	111	0.435

**Table 3:** Univariate Cox proportional hazards regression analysis for the predictors of MACCE.

Characteristic	HR	95% CI	*p*-Value
Age (years)	1.124	1.04, 1.214	0.0031 *
Sex (Ref: Female)			
Male	1.528	0.2552, 9.153	0.642
MSC IV dose (in billions)	0.667	0.203, 2.191	0.504

Abbreviations: CI, confidence interval; HR, hazard ratio. * indicates statistical significance.

**Table 4:** Multivariate Cox proportional hazards regression analysis for the predictors of MACCE.

Characteristic	HR	95% CI	*p*-Value
Age (years)	1.127	1.0418, 1.219	0.0029 *
Sex (Ref: Female)			
Male	0.9129	0.1429, 5.831	0.9232
MSC IV dose (in billions)	0.5928	0.1849, 1.900	0.379

Abbreviations: CI, confidence interval; HR, hazard ratio. * indicates statistical significance.

## Data Availability

All data generated or analyzed during this study are included in this article.
